# Recommended long COVID outcome measures and their implications for clinical trial design, with a focus on post-exertional malaise

**DOI:** 10.1016/j.ebiom.2025.106083

**Published:** 2025-12-19

**Authors:** Letícia Soares, Hannah Davis, Ezra Spier, Tiffany Walker, Todd Davenport, David Putrino, Michael Peluso, Julia Moore Vogel

**Affiliations:** a Patient Led Research Collaborative, United States; b Department of Medicine, Emory University, Atlanta, GA, 30322, USA; c University of the Pacific, Stockton, CA, USA; d Mount Sinai School of Medicine, New York, NY, USA; e University of California San Francisco, San Francisco, CA, USA; f Scripps Research Translational Institute, Scripps Research, La Jolla, CA, 92037, USA

**Keywords:** Long COVID, Endpoints, Outcomes, Clinical trials, Biomarkers

## Abstract

Long COVID has created a worldwide public health crisis and has no approved treatments or validated biomarkers. We summarize the current challenges and considerations of outcome selection in Long COVID trials, along with recommendations for current trial design and future endpoint validation, with a focus on post-exertional malaise (PEM). We make five overarching recommendations for Long COVID clinical trials: 1) thorough characterisation of baseline disease; 2) collection of longitudinal data; 3) design of a placebo arm to enable comparison of treatment effect relative to the disease natural history; 4) accounting for, and when feasible, measuring PEM; 5) balancing severity, duration, and relevant phenotypes across trial arms and within subgroups to be analysed. We present a list of outcomes that may be considered for Long COVID clinical trials, with a focus on PEM. Crucially, the field of Long COVID clinical trials urgently needs funding and research effort investment to develop and validate outcomes concomitantly with clinical trial research.

## Introduction

Long COVID is a worldwide public health crisis that has affected over 400 million individuals^1^ with no approved treatments. While there are numerous options to manage Long COVID symptoms, there are no treatments to address the root cause.^2^, ^3^, ^4^ Several recent trials have produced negative results^5^, ^6^, ^7^, ^8^, ^9^; note that reference 7 has not been peer reviewed. Lack of validated endpoints for Long COVID may discourage funders and drug developers from supporting additional Long COVID clinical trials. Given the variability in its clinical presentation array of underlying mechanisms, the difficulty of capturing key components such as post-exertional malaise (PEM), and a rapidly evolving research base, there is unlikely to be one outcome that can capture the scope of Long COVID's progression or resolution. We specifically focus on disease features that are underresearched and challenging to study, and that strongly affect patient outcomes and disease severity, namely disease heterogeneity and natural history, as well as post-exertional malaise (PEM) and phenotypes that include myalgic encephalomyelitis/chronic fatigue syndrome (ME/CFS). We believe these factors to be the most challenging to address in Long COVID clinical trial design.

## Heterogeneous presentation and natural history

In Long COVID, the affected systems, new-onset conditions, intensity, and impact on quality of life differ significantly between individuals.^10^^,^^11^ Several mechanisms are hypothesised to contribute to the physiological disruptions in Long COVID, including autoimmunity and other forms of immune dysregulation, dysfunctional neurological signalling, gut dysbiosis and translocation, inflammation, latent virus reactivation, mitochondrial dysfunction, thrombosis, and viral persistence.^2^^,^^4^ Within an individual, symptom profiles can evolve over time, and factors such as environmental changes, medications, SARS-CoV-2 reinfections, and hormonal fluctuation can influence symptom presentation.^4^^,^^12^ We recommend using multiple measurements to thoroughly characterise baseline disease (Table 1). In addition, analyses should include the number of SARS-CoV-2 infections. Further, it may take time for interventions to reach their full impact, and impact may be temporary. Single time-point assessments, even when expressed as change from baseline, fail to capture these dynamics, as do studies that conclude too early. Collecting and utilizing longitudinal data to more fully quantify the impact of treatments is crucial. To longitudinally control for the natural history of the disease, we recommend using a placebo arm, and whenever suitable, apply a controlled crossover design. Based on the intervention and its mechanism of action, investigators should consider targeting specific subsets of participants, such as those with a specific new-onset condition (e.g. dysautonomia) or debilitating symptom (e.g. cognitive dysfunction), as individuals with shared symptomatology may also have shared underlying pathobiology.

**Table 1:** List of endorsed outcomes for Long COVID clinical trials, accompanied by rationale, use and applicability limitations, as well as research needs to propel the field forward.

Outcome(s)	What it measures	Rationale	Limitations	Needs
Cerebral blood flow (upright) measured by transcranial doppler^13^	Dysautonomia	Reduced cerebral blood flow was detected among 82% of people with ME/CFS with normal HR/BP response to head-up tilt test (N = 247)^14^	Requires specialized equipment and training of personnel.	Baseline longitudinal characterisation.
Standard Autonomic Functioning Test (evaluation of cardiovagal, sudomotor and adrenergic autonomic functions^15^)	Dysautonomia	Can detect subclinical dysautonomia.^16^ Tilt table testing can fail to detect orthostatic intolerance in Long COVID; it occurs more often among people who have had Long COVIDfor longer than one year.^17^	Requires specialized equipment and personnel.	
10-min tilt table test (with beat-to-beat blood pressure and continuous heart rate monitoring^15^)	Dysautonomia	Differentiates tachycardia due to POTS, and can characterise orthostatic hypotension.^18^	Requires specialized equipment and personnel, intentionally exacerbates symptoms and must be done carefully, with informed consent.^19^^,^^20^	
Cognitive assessment (e.g. CNS Vital Signs, Cogstate)	Cognitive function	Cognitive dysfunction is commonly experienced. Decreased cerebral blood flow is associated with cognitive dysfunction.		Validation in the context of Long COVID.
DSQ-PEM and DSQ-PEM-SF^17^	Determines whether the patient's phenotype includes PEM.	PEM scoring thresholds were met by 58.7% of 213 people with Long COVID.^21^	Not designed to assess ongoing PEM.	Design and validation of a companion instrument that captures PEM as it is occurring.
Question enquiring whether PEM is present at the time of sampling.	Ongoing PEM	The presence of PEM may affect other outcome measures, and should be accounted for in statistical analyses.	Many people who experience PEM practice pacing to prevent and manage PEM. Measures may be associated with an individual's effectiveness at pacing.	Validation in the context of Long COVID.
2-day CPET	PEM by quantifying impaired exercise capacity in response to exertion,^22^ with hand grip strength as an alternative for severe patients and low resource environments.^23^^,^^24^	Compared to healthy and sedentary controls, impaired functional capacity in response to exercise-induced exertion has been extensively documented in ME/CFS.^25^, ^26^, ^27^, ^28^	Requires specialized equipment and training of personnel. Not accessible to people with severe Long COVID. Induces PEM, thus benefit to risk ratio must be carefully assessed. On average, people with ME/CFS take two weeks to recover from a 2-day CPET.^29^	Wider embrace of 2-day CPET as an endpoint to determine effects on metabolism, symptoms, and functioning. Use of 2-day CPET to validate other tools to better characterise the pathophysiology and functional effects of PEM.
PEM/PESE Activity Questionnaire (PAQ)^18^	PEM related disability	PAQ can be used to longitudinally assess variation in functional capacity in response to PEM.	Not suitable for assessment of functional capacity in patients who do not experience PEM.	
Fatigue Severity Scale, Multidimensional Fatigue Inventory	Fatigue^30^^,^^31^	Fatigue is one of the most common Long COVID symptoms. Fatigue is not necessarily a component of PEM. Fatigue and PEM must be assessed independently.	May not be sensitive to changes in symptoms. Fatigue alone may not distinguish people with Long COVID from people with other fatiguing illnesses. May have ceiling effects for people with ME/CFS.^32^	Development and validation of a fatigue instrument that captures and measures dimensions of fatigue in Long COVID (e.g. physical, cognitive^33^).
FUNCAP-27 or FUNCAP-55^34^	Functional Capacity	Designed to assess functional capacity in ME/CFS while directly accounting for PEM. Effectively distinguishes functional capacity levels between people with mild, moderate, severe, and very severe illness.		Needs validation for Long COVID.
Physical function subscale of the SF-36 or EQ-5D-5L	Quality of Life	SF-36 or EQ-5D-5L are recommended when one seeks to compare quality of life to other illnesses.	SF-36 does not discriminate well across disease severity due to floor effects.^32^	PRO assessing health-related quality of life specifically for Long COVID.
PHQ-2 or instruments to assess mental health that do not rely on somatic symptoms frequently reported in LC	Mental Health	Eliminates the risk of inflating the instrument's score by avoiding conflating fatigue with depression, and autonomic dysfunction symptoms with anxiety.	The majority of people with Long COVID do not have a mental health condition.^35^	
SARS-CoV-2 RNA: ddPCR, qPCR; or SARS-CoV-2 Antigen: SIMOA	SARS-CoV-2 persistence	It is uncommon for nucleic acid tests to identify SARS-CoV-2 persistence in the blood of people with Long COVID. We recommend either being aware of the false negatives that are expected when sampling blood and/or conducting tissue biopsies in likely reservoir locations (e.g. colon, lymph node, muscle, reproductive tract, nerve).		
EBV panel alongside Early Antigen; other herpesvirus tests; bacteria.	Reactivation of other viruses and common bacterial infections.	Reactivation of herpesviruses (particularly EBV and HHV-6) is common after SARS-CoV-2 infection, and clinicians report that tick-borne infections (e.g. Borrelia, Bartonella, Babesia) are commonly detected in patients with Long COVID.	Standard lab tests for bacteria are not very sensitive in people with long-term infections, so should be used with caution.	While these markers are particularly promising, they need further validation.
Microclots; Platelet hyperactivation; EndoPAT; Perfusion index and SpO2; Blood vessel morphology and size; Retinal/corneal markers^36^^,^^37^	Endothelial and/or vascular dysfunction	Endothelial dysfunction is characterised as a downstream pathophysiology of Long COVID.^2^^,^^4^^,^^38^		While these markers are particularly promising, they need further validation.

## PEM

PEM is a clinical hallmark of ME/CFS, which is a key phenotype of Long COVID.^39^^,^^40^ It is the aspect of Long COVID that has the most substantive impact on clinical trial design and the capability to include patients with severe Long COVID. PEM is a complex pathophysiologic state resulting from exertion (physical, cognitive, sensory, and emotional) and environmental triggers.^41^, ^42^, ^43^ Symptoms of PEM include, but are not limited to, debilitating fatigue, cognitive, gastrointestinal and sleep dysfunction, and sensory sensitivity. PEM is difficult to measure, and may be counterintuitive to those who lack familiarity with the condition. PEM causes a person's abilities to vary substantially within hours to days (“microcycling”) and within weeks to months (“macrocycling”).^11^^,^^44^^,^^45^ There is often a delayed onset between the trigger and PEM, which may vary based on the individual, the trigger itself, and the length the patient has been ill.^10^^,^^46^^,^^47^ PEM is also characterised by prolonged and often incomplete recovery, which may reduce the overall baseline for functional activities over time.^42^

Any study that includes people who experience PEM must measure it because PEM can induce variability. PEM is a substantial challenge for endpoint selection, underscoring the importance of collecting whether or not a patient is experiencing PEM during each measurement. Symptom variability is influenced by the extent to which people who experience PEM can prevent it, therefore activity level should be considered. Any measurements taken as part of a trial could trigger or aggravate PEM, potentially hindering data collection and increasing participant risk. Tests that induce PEM without measuring it or accounting for baseline fluctuation in functional capacity–such as the 6-min walk test and the 1-min sit-to-stand test–may not be appropriate as outcomes for Long COVID clinical trials, and should only be used to enable comparisons with other diseases.

DSQ-PEM is a validated instrument we recommend to screen for PEM based on patient-reported experiences.^48^ However, DSQ-PEM may not be optimal for assessing changes in PEM over time, including with regard to the study activities (which could exacerbate PEM) or the study intervention (which may aim to reduce PEM). A patient reported outcome (PRO) that captures a snapshot of PEM status and symptoms needs to be developed and validated for people with Long COVID. Until such an instrument is available, we recommend educating participants about PEM, asking whether participants are currently experiencing PEM at each data collection point and analysing these data as a covariate.

Sometimes, researchers may aim to objectively quantify PEM. The gold standard for doing so is a 2-day cardiopulmonary exercise test (CPET)—an approach that has been used in ME/CFS since prior to the pandemic^40^^,^^22^^,^^25^ and to suggest potential treatments.^26^ It uses an initial CPET to measure baseline performance and as a method to induce PEM, and a second CPET to measure the physiological and perceptual responses to exercise during PEM. The use of a single CPET is not recommended, as the first CPET does not capture the PEM state.^27^^,^^49^ The downsides of CPET methodology are that it involves more equipment and training than PROs, comes with risk to participants given that it triggers PEM, requires the participant to travel to the equipment, and is inappropriate for people with severe/very severe PEM. In studies in which CPET will be performed, participants must be thoroughly informed of the risks associated with CPET during the informed consent process.^22^ We conditionally recommend using 2-day CPET, depending on feasibility (costs, accessibility, and participation burden) and on trial design (objectives and endpoint selection). Emerging measurable pathophysiological changes associated with PEM induced by an exercise challenge include tissue damage, skeletal muscle necrosis, tissue infiltration of amyloid-containing deposits, and mitochondrial dysfunction; however, further validation of these methods is needed.^50^

## Symptom severity, illness duration and phenotypes

Using severity and improvement measurements as outcomes is challenging due to substantial variability in natural history across Long COVID phenotypes. For example, people with Long COVID without new-onset ME/CFS are more likely to experience resolution of dysautonomia, improvement in pain, and decreased illness severity compared to those with new-onset ME/CFS.^51^ Different symptom presentations have varying likelihoods of improvement based on illness duration,^52^ with neurological and cognitive presentations being particularly unlikely to improve or even increase in severity over time.^51^^,^^53^ Sex may impact improvement likelihood, with males being more likely to recover than females.^34^, ^51^ We recommend balancing the sample, including across randomised arms, for relevant comorbidities associated with phenotypes of interest, illness severity, illness duration, and sex.

## Biomarkers as endpoints, and challenges with illness duration

At least 239 potential biomarkers have been proposed for Long COVID, of which just 8% have been analysed for receiver operating characteristics.^54^ Promising potential biomarkers include autoantibodies, circulating microclots, inflammation markers, and viral persistence.^2^^,^^55^ One challenge to biomarker validation is that their pattern may change with illness duration. For example, proinflammatory markers that are elevated after acute infection tend to decrease over time and be uncorrelated with symptom burden.^56^, ^57^, ^58^ Illness duration has also been identified as a major factor in ME/CFS where cytokine profiles differ between those who have had the condition for under versus over three years.^59^ Illness duration in ME/CFS can also affect the efficacy of response to therapeutic interventions.^60^ Thus, it is crucial to balance illness duration subgroups within control and intervention cohorts.

Examining biomarkers associated with the intervention's mechanism of action, e.g. specific upstream or downstream pathophysiology,^2^ can improve understanding of its use in Long COVID. As understanding of the pathophysiology of Long COVID evolves, biomarkers may clarify disease mechanisms and phenotypes, guide phenotype-specific treatments, and refine endpoints for targeted therapies.

## Secondary endpoints

Because the field's endpoints are still being developed and validated, we recommend including secondary endpoints when possible. Comparing secondary endpoints under development to those in use can enable exploration of endpoint correlation, including promising biomarkers, and condition-specific tests for components such as cognitive dysfunction or dysautonomia. Using secondary endpoints will advance endpoint development and strengthen the field.

## Development and validation of PROs

Significant financial investment and research effort are needed into developing PROs for Long COVID. Whether a PRO is repurposed or newly derived, it will require validation within the context of Long COVID. Validation requires demonstrating the measure is relevant across the spectrum of disease, that it is specific to the detection of the disease and sensitive to change in the disease state, and that it is working as intended. Traditionally, this is done prior to trial start. In a pandemic-related disease, we recommend that trialists embed validation procedures into early data collection. Where tools can be translated to Long COVID, questionnaires applied and validated in the infection-associated chronic illness patient population can provide a firm foundation.^61^, ^62^, ^63^

New PRO endpoints should be designed to accommodate varying levels of health literacy, digital access, language proficiency, and disability. We recommend having new survey instruments co-created with people with lived experience and reviewed by individuals with diverse cultural backgrounds to improve cultural competency. Global scalability of new PRO endpoints will require cross-cultural adaptation.

Until Long COVID phenotype associations are characterised and PROs are validated, global PROs are preferable. One global PRO that shows promise as an endpoint for Long COVID is the FUNCAP.^64^ FUNCAP is a validated tool to measure functional capacity considering PEM and fluctuations in capacity across eight domains, and is sensitive to changes across severities. People with severe Long COVID can be included using a combination of patient-centred PROs such as FUNCAP, and accessible clinical and biomarker outcomes. Limitations of using global PROs as primary endpoints include the risk of diluting a treatment effect into nonsignificance, and loss of explanatory capacity over what the treatment is improving.

## Conclusion

While there is an urgent need for PRO and biomarker validation to strengthen endpoints for Long COVID clinical trials, trials that are conducted in the interim can increase their potential for success by following the best practices outlined herein.

## Contributors

LS, HD, ES, and JMV conceptualised the manuscript and wrote the initial draft. All authors (LS, HD, ES, TW, TD, DP, MP, and JMV) contributed to editing the initial version, reviewing the final version, and approved its submission.

## Declaration of interests

LS has received consulting fees as a patient-advisor to London School of Economics and Political Sciences, University of Toronto, Oswaldo Cruz Foundation, and Food and Drug Administration. HD has received consulting fees as a patient-advisor to Vanderbilt University, CureID, NIH RECOVER, and N3C. ES has received contract support as a patient-advisor to BioVie and consulting fees to NIH RECOVER. TW has no interests to declare. TD has received occasional honoraria and travel costs for speaking at conferences and educational events, as well as volunteering as a scientific advisor to Workwell Foundation. DP has received grants from Polybio Research Foundation and Steven and Alexandra Cohen Foundation. MP has received grants from NIH, Polybio Research Foundation, and Patient Led Research Collaborative along with consulting fees from Gilead Sciences, AstraZeneca, Apellis Pharmaceuticals, BioNTech, and BioVie, and travel support from Invivyd. JMV has received consulting fees as a patient-advisor from Sage Bionetworks and NIH RECOVER.
